# AI outperformed every dermatologist in dermoscopic melanoma diagnosis, using an optimized deep-CNN architecture with custom mini-batch logic and loss function

**DOI:** 10.1038/s41598-021-96707-8

**Published:** 2021-09-01

**Authors:** Tri-Cong Pham, Chi-Mai Luong, Van-Dung Hoang, Antoine Doucet

**Affiliations:** 1 ICT Laboratory, Vietnam Academy of Science and Technology, University of Science and Technology of Hanoi, Hanoi, 100000 Vietnam; 2 Thuyloi University, 175 Tay Son, Dong Da, Hanoi, 10000 Vietnam; 3 FPT Software, 17 Duy Tan Street, Cau Giay district, Ha Noi, 10000 Vietnam; 4 Institute of Information Technology, Vietnam Academy of Science and Technology, Hanoi, 100000 Vietnam; 5 Ho Chi Minh City University of Technology and Education, Ho Chi Minh, 510000 Vietnam; 6 L3i Laboratory, University of La Rochelle, 17000 La Rochelle, France

**Keywords:** Computer science, Cancer screening

## Abstract

Melanoma, one of the most dangerous types of skin cancer, results in a very high mortality rate. Early detection and resection are two key points for a successful cure. Recent researches have used artificial intelligence to classify melanoma and nevus and to compare the assessment of these algorithms to that of dermatologists. However, training neural networks on an imbalanced dataset leads to imbalanced performance, the specificity is very high but the sensitivity is very low. This study proposes a method for improving melanoma prediction on an imbalanced dataset by reconstructed appropriate CNN architecture and optimized algorithms. The contributions involve three key features as custom loss function, custom mini-batch logic, and reformed fully connected layers. In the experiment, the training dataset is kept up to date including 17,302 images of melanoma and nevus which is the largest dataset by far. The model performance is compared to that of 157 dermatologists from 12 university hospitals in Germany based on the same dataset. The experimental results prove that our proposed approach outperforms all 157 dermatologists and achieves higher performance than the state-of-the-art approach with area under the curve of 94.4%, sensitivity of 85.0%, and specificity of 95.0%. Moreover, using the best threshold shows the most balanced measure compare to other researches, and is promisingly application to medical diagnosis, with sensitivity of 90.0% and specificity of 93.8%. To foster further research and allow for replicability, we made the source code and data splits of all our experiments publicly available.

## Introduction

Melanoma is one of the most dangerous skin cancers, which accounts for the majority of skin cancer deaths. The detection of the disease starts with the visual examination of skin lesion images by dermatologists, and more pathological analyses will be performed if there is a suspicion. If detected early, the cancer can be cured up to 95% of the cases^1^ . Computer vision in the field of artificial intelligence (AI) has recently achieved very excellent results in image recognition, even exceed humans in some problems with large datasets^2^. This inspires many studies about AI-based solutions for automating melanoma diagnosis using skin lesion images, especially using deep Convolution Neural Networks (CNN) for the melanoma prediction problem^3–7^. Still, there are challenges due to the limited data and the imbalance between melanoma and non-melanoma images in the datasets. In 2019, the largest public skin lesion image dataset, the ISIC 2019^1^ with 25,331 dermoscopic images of eight different categories, was released. This dataset, together with the MClass-D released by Brinker et al.^7^ that consists of 100 dermoscopic images and diagnosis results from 157 dermatologists, make possible for researchers to train their AI models with large data and provide a benchmark for performance comparison.

One of the biggest challenges when tackle deep learning problems on medical image is imbalanced dataset. When a neural network is trained on datasets that have one or more classes extremely low in the number of samples, the model performs poorly on the minority classes^8^. This is shown by the loss of the minority class improving very little through iterations while that of the majority class is significantly improved^8^ . The two approaches to solve this problem are data level methods (re-sampling) and algorithm level methods. The re-sampling methods change the proportion of classes by increasing the number of samples of the minority classes or reducing the number of samples of the majority classes so that the ratio of classes is balanced in the training dataset. This method is simple and easy to implement. Meanwhile, algorithm level methods are more difficult and more complex because we have to devise algorithms to impact the training process for more balanced performance even when models are learned with imbalanced dataset. One simple method is re-weighting losses of examples during training. The loss weights can be fixed per class^9^ , per example, or based on the cost-sensitive of the misclassified examples^10–12^. Unfortunately, in many domains, it is very hard to define the cost and we only have known that the cost of misclassified samples of the minority class is higher than that of the majority class^13^.

In the research of deep learning for skin cancer problems, there have been studies on re-weighting methods such as changing weights for losses, increasing weights for examples that have been verified by biopsy^9^ , but there have not been any studies on how to change the loss function so that the model achieves a higher generality for problems with imbalanced data. Therefore, in this study, we propose a combination of custom loss function and custom mini-batch logic to optimize neural networks for the melanoma classification problem and have the following contributions.

1. Proposing a custom loss function (CLF) to handle imbalanced datasets of melanoma and nevus. The CLF is calculated by the separate loss of positive mean squared error on the melanoma images and negative mean squared error on nevus images. The CLF improves the learning ability of the neural networks on the minority class of melanoma. Thus, it enhances the balance performance of the models which is evaluated by sensitivity (SEN) and specificity (SPE) as melanoma prediction problems.
2. Proposing a custom mini-batch logic followed by a real-time image augmentation for enhancing the effect of the CLF when training neural networks on imbalanced datasets. Our proposed custom mini-batch logic ensures the proportion of melanoma and nevus in every mini-batch of the training.
3. Proposing a solution, which combines the CLF and custom mini-batch logic in an optimized deep CNN architecture, for melanoma prediction. The solution is evaluated by comparing with the performance of dermatologists on the same MClass-D dataset^7^.

## Optimized melanoma prediction system

### Proposed melanoma prediction system

**Figure 1:**
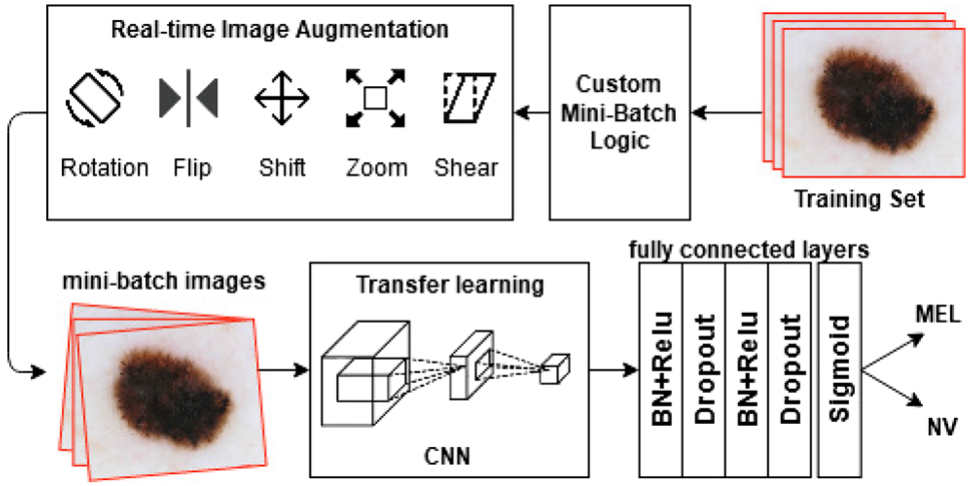
Proposed melanoma prediction system (this figure was created with Draw.io).

In this research, we propose a melanoma prediction system consists of four main components: Custom mini-batch logic, Real-time Image Augmentation, CNN and Reformed fully connected layers as illustrated in Fig. 1.

**Figure 2:**
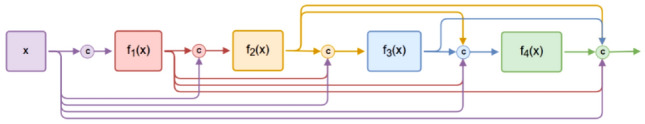
Dense connections in DenseNet (this figure was created with Draw.io).

In general, a number of skin lesion images are selected from the training dataset by custom mini-batch logic module. This batch logic picks training images into batches in a way that the proportion between classes is fixed. After that, the selected images are augmented by the Real-time Image Augmentation module and then put through a CNN to extract salient features. The extracted features are then gone through customized fully connected layers to classify whether the skin lesion image is melanoma or not. Also, to increase the learning ability of the networks and to improve efficiency of our solution on the melanoma dataset, we optimize the added fully-connected layers with batch normalization (BN) followed by dropout layers. The output layer uses sigmoid activation function for binary classification. This neural network is trained with our proposed custom loss function of two classes detailed Fig. 2.

#### Selecting CNN architecture

We experiment with several outstanding CNN architectures such as InceptionV3^14^, ResNet50^15^, DenseNet169^16^. We use transferred CNNs which are trained by ImageNet to re-train with normal batch logic and common loss function, then compare the results, and select the best architecture for melanoma prediction. However, the problem of InceptionV3 is underfitting; it does not always converge during training; AUC is low along with SEN and SPE has a large deviation (showed in Table 1). As for ResNet50, the training process converged, but its SEN, SPE still has a large deviation and AUC is low on Test-10 and MClass-D. The DenseNet169 obtains the best result of all architectures on the three measures of SEN, SPE and AUC. This is because of the key differences between DenseNet and the other 2 models. As shown in Fig. 2, key ideas of DenseNet are (1) to use the input of each layer to later be part of its output and (2) instead of using additional functions as ResNet50 does, DenseNet uses a concatenation function to calculate the output from x and f(x). These two features explain why this approach is adequate for image classification with images containing few and large objects, as the binary melanoma classification task.

**Table 1:** Performance of original InceptionV3 of melanoma prediction.

Loss function	Test-10			MClass-D		
	AUC	SEN	SPE	AUC	SEN	SPE
Binary cross entropy	90.6	61.8	96.1	84.0	55.0	95.0
Focal loss	89.7	46.2	98.6	85.4	35.0	98.8
Mean square error	87.5	73.1	84.2	89.8	80.0	83.8

### Custom loss function for imbalanced datasets

This research designs a custom loss function for addressing the imbalanced class problem of melanoma and nevus. A deep CNN learns from data by iteratively calculating the loss and update the weights of the layer’s nodes accordingly so that the loss is gradually minimized^2^. This process depends a lot on the loss function because it tells the model how badly the model predicts and how much to update each node’s weight so that the model can improve itself. Some loss functions commonly used in research are mean squared error, binary cross entropy, squared hinge, sparse categorical cross entropy, categorical cross entropy, categorical hinge, etc. These loss functions have the same final logic that at each iteration of training, they are the sum, over all the data samples, of the loss calculated by the difference between the predicted and actual target label, divided by the number of samples^2^. The neural networks are trained by using these functions on a balanced dataset to perform high accuracy (ACC), the metric is calculated by the rate of the number of properly predicted samples divided by the total number of samples.

However, applying these functions to melanoma prediction on an imbalanced dataset is not effective because when the backpropagation algorithm updates the weights of neural networks on an imbalanced dataset^8^, the process is impacted significantly by the majority class. This leads to an imbalanced performance between SEN and SPE (low SEN versus SPE), while they are the most important evaluation metrics of the medical image classification problems. Thus, we need to optimize SEN and SPE separately by using custom loss function^17^.

In this study, we propose a custom loss function (CLF) based on the separate loss of positive mean squared error on the melanoma images and negative mean squared error on nevus images. These below formulas explain loss functions for: (1) mean squared error (namely $l_{MSE}$); (2) positive mean squared error on the melanoma images (namely $l_{PMSE}$); (3) negative mean squared error on nevus images(namely $l_{NMSE}$); and (4) our proposed CLF (namely $l_{CLF}$):

1 $$\begin{aligned} l_{MSE}= & {} \frac{\mathrm 1}{\mathrm M}\displaystyle \sum _{i=1}^{M} (y_{i} - y^{*}_{i})^{2} \end{aligned}$$

2 $$\begin{aligned} l_{PMSE}= & {} \frac{\mathrm 1}{\mathrm P}\displaystyle \sum _{i=1}^{P} (y_{i} - y^{*}_{i})^{2} \qquad ,with\ y_{i}=1 \end{aligned}$$

3 $$\begin{aligned} l_{NMSE}= & {} \frac{\mathrm 1}{\mathrm N}\displaystyle \sum _{i=1}^{N} (y_{i} - y^{*}_{i})^{2} \qquad ,with\ y_{i}=0 \end{aligned}$$

4 $$\begin{aligned} l_{CLF}= & {} a \times (l_{PMSE} + l_{NMSE})^{2} + b \times (l_{PMSE} - l_{NMSE})^{2} \end{aligned}$$

In a dataset of *M* samples, *N* is the number of negative samples (nevus), *P* is the number of positive samples (melanoma). Then, *M=N+P*. $y_{i}$ and $y^{*}_{i}$ is the ground truth and predicted label of the *i*th sample. The Formula (1) presents mean squared error on entire dataset, $l_{MSE}$ is calculated by average loss of *M* samples in the entire dataset. When this loss is zero that means our model achieve best performance on entire dataset. When we apply mean squared error on positive subset of melanoma images, we have Formula (2), $l_{PMSE}$ is the $l_{MSE}$ apply on a subset of *P* melanoma images and Formula (3), $l_{NMSE}$ is $l_{MSE}$ apply on a subset of *N* nevus images. This means that minimizing $l_{PMSE}$ and $l_{NMSE}$ leads to maximum SEN and SPE respectively. Designing Formula (4) of the *CLF* aims at increasing $|SEN + SPE|$ along with decreasing $|SEN - SPE|$ to zero. This is based on three main ideas.

1. The *CLF* is depend on ($l_{PMSE} + l_{NMSE}$) to optimize $|SEN + SPE|$.
2. The *CLF* is depend on ($l_{PMSE} - l_{NMSE}$) to optimize $|SEN - SPE|$.
3. We square ($l_{PMSE} + l_{NMSE}$) and ($l_{PMSE} - l_{NMSE}$) to avoid positive and negative values canceled out each other. Besides, we add *a* and *b* coefficients as adjustable parameters.

In this study with limited resources, we use the Trial and error method to observe a and b coefficients to achieve high performance in order to illustrate that the *CLF* is able to increase the learning ability of the neural networks on the imbalanced melanoma dataset. The b coefficient should be larger than a to make the penalty worse when the model has too much difference between these two metrics. The final selection of a and b in this research is *a=0.5* and *b=1.0*.

To understand the effect of the *CLF*, we illustrate three examples of confusion matrix on a 100-image dataset includes 20 images are positive (minority class) and 80 images are negative (majority class), which is the same class proportion of the MClass-D. The value of *CLF* is calculated with *a* = 0.5 and *b* = 1.0. The ACC, SEN, SPE, $l_{MSE}$, and $l_{CLF}$ of three examples are demonstrated in Table 2.

**Table 2:** Confusion matrices and loss values of three examples, in terms of SEN and SPE, the best is Example 3 and the worst is Example 1.

Predicted class		True class		ACC	SEN	SPE	$l_{MSE}$	$l_{CLF}$
		P	N					
1	P’	12	2	90.0	60.0	97.5	0.10	0.2309
	N’	8	78					
2	P’	16	8	88.0	80.0	90.0	0.12	0.050
	N’	4	72					
3	P’	17	10	87.0	85.0	87.5	0.13	0.0384
	N’	3	70					

In Table 2, from Example 1 to Example 3, the (SEN + SPE) increases and (SPE − SEN) decreases, while ACC decreases. Along with that, MSE increases while CLF decreases. This indicates that CLF is the better loss function than MSE to optimize SEN and SPE on an imbalanced dataset. In more detail, Example 3 perfectly reflects what we aim to achieve since it has the best performance of SEN combined with SPE and the lowest loss at 0.0384. This advantage is demonstrated and analyzed in “Evaluation of loss, sensitivity and specificity during training and validation”.

### Custom batch logic and real-time image augmentation

Training deep CNN requires a large number of samples in the dataset. Therefore, at each iteration of the training, it cannot be optimal on the entire data because such computation is complex and resource consuming. In order to optimize the training, the dataset is divided into smaller subsets using batch logic, then at each iteration, a subset is used instead of the entire dataset. Images of a subset are randomly selected leads to a dynamic distribution of the majority and minority classes in each iteration, especially when compared to their distribution in the entire dataset. Thus, the number of minority images (NPI) in an iteration can be too many, equal, and most of them too few (some cases is zero) compared to the number of majority images (NNI). Having an NPI greater than, equal to or near an NNI reduces the effectiveness of the *CLF*. In these cases, the *CLF* trains the neural network to learn the majority class faster than the minority class. Besides, losing lots of samples for these cases result in increasing the number of iterations have too few or even zero NPI. In these iterations, the learning of the neural network is inefficient on the minority class either. In this study, we propose a custom mini-batch logic with the following characteristic and advantages.

1. The proportion of majority and minority classes in a mini-batch of training is unchanged, the ratio of NPI/NNI is fixed in every iteration.
2. Each sample of the same class is equally effective in teaching neural networks at each iteration of training.
3. At each iteration of training, each sample of the minority class is more impactful than that of the majority class because the *CLF* is used for training the neural networks.

This proposed custom mini-batch logic is combined with real-time augmentation to prepare training data before being extracted features by the CNN. Unlike offline augmentation where entire dataset is augmented then randomly selected later for training batch, real-time augmentation generates images when they are selected. This ensures images in a batch do not all come from the same original image, which could happen with offline augmentation. This improves the effect of training the neural networks, especially when combined with the CLF loss function. In this research, the images are selected by custom mini-batch logic to ensure the proportion of melanoma and nevus classes the same in every iteration of training. Then, the images are augmented before extracted features by convolutional layers. When combined with the CLF loss function, this process enhances the learning effectiveness on both minority and majority classes. This advance result is illustrated and analyzed carefully in “Evaluation of loss, sensitivity and specificity during training and validation”. In this research, we implement augmentation, based on specialists knowledge, by the combination of the following transforms with random in a period of: (1) Image rotation within the range of 5$\circ$; (2) Simultaneously, image shifting within the amplitude range of 0.05%, specifically, to the right and left within the angle range of 0.05%, up and down within the angle range of 0.05%; (3) Image shear within the range of 0.05$\circ$; (4) Image zoom in and out within the range of 0.05%; (5) Lastly, applying fill mode using algorithm ‘nearest’;

### Reform architecture of fully connected layers

To take advantage of the CLF function, custom mini-batch logic, and real-time image augmentation, we need an appropriate deep CNN, and especially the fully connected layers must have the ability to learn on the large dataset of around seventeen thousand images which are also augmented many times. In this study, we reform the architecture of fully connected layers with the following features.

1. Customized architecture has two hidden layers that use ReLU as the activation function.
2. The first layer has 1024 nodes and the second layer has 512 nodes.
3. Both hidden layers use Batch normalization and Dropout to improve the efficiency of our solution.
4. The output layer with sigmoid activation function is applied for binary classification.

#### Dropout

Dropout is a simple and effective regularization technique which randomly ignores a certain number of nodes in the neural networks during training to prevent overfitting. Thus, it improves the performance of deep neural networks over other regularization methods^18^. In our system, a dropout block with a rate of 0.5 is added after each hidden layer.

#### Batch normalization

Batch normalization normalizes the inputs to a network by subtracting the batch mean and dividing by the batch standard deviation. We apply batch normalization^19^ in each hidden layer to improve the stability of a neural network.

## Experimental materials

### Datasets

In this research, we use the ISIC 2019^1,20–22^ challenge dataset including 25,331 dermoscopic images across eight different categories to train and evaluate our proposed algorithm. This dataset is the latest public dataset about skin cancer. This study focuses on the classification of melanoma and nevus, thus we use only images with types of melanoma and nevus in the ISIC 2019. As shown in Table 3, after removing unrelated images, the remaining images used in the study are 17,302 images including 4503 melanoma images (minority class) and 12,799 nevus images (majority class). The images are in high resolutions, can be approximately up to 1800 $\times$ 1200 pixels. These images are center cropped, resized to the size of 256 $\times$ 192 px, and then normalized by adding multiples a converting all pixels into [0, 1.0] range. We use 80% of the images (13,842 images including 3603 melanoma images and 10,239 nevus images) as training data (namely Train dataset), 10% of the images (1730 images including 450 melanoma images and 1280 nevus images) as validation data (namely Validation dataset) and the remaining 10% of the images (1730 images including 450 melanoma images and 1280 nevus images) as testing data (namely Test-10 dataset).

An important goal of this research is to design a Deep CNN architecture that is more effective than the state-of-the-art binary melanoma classification approaches, including the direct assessment by dermatologists. Thus, we use the MClass-D dataset of Titus J. Brinker et al.^7^ to evaluate our model. This dataset contains 100 dermoscopic images (20 melanoma images, 80 nevus images). Furthermore, the images of MClass-D are also selected from the ISIC archive, therefore, images that also belong to MClass-D are removed from the ISIC 2019 dataset before being split into the Train, Validation, and Test-10 dataset as mentioned above.

**Table 3:** Melanoma train, validation, test, and benchmark datasets.

Resources	Type	Melanoma	Nevus	Total
ISIC 2019	Train	3603	10,239	13,842
ISIC 2019	Validation	450	1280	1730
ISIC 2019	Test	450	1280	1730
The Benchmark	MClass-D	20	80	100
Total of ISIC 2019		4503	12,799	17,302

### Dermatologist performances

To compare the performance of artificial intelligence automated diagnosis with human dermatologists, Brinker et al. send 100 skin lesion images to 157 dermatologists at different German university hospitals and receive individual results per dermatologist per image. Then they evaluate the doctor’s performance in terms of area under the curve (AUC), SEN, and SPE. This dataset enables researchers to compare their algorithm performances with that of dermatologists. Summary information about the diagnosis results of 157 dermatologists is described in Table 4. The SEN and SPE of individual dermatologists are also illustrated by red dots in Figs. 7 and 9 to compare with our proposed solution.

**Table 4:** Diagnostic performance of 157 dermatologists by MClass-D dataset.

Subset of dermatologists	AUC	SEN	SPE
**All participants (n = 157)**	67.1	74.1	60.0
University hospital (n $=$ 151)	66.9	74.0	59.8
Private practice (resident) (n $=$ 6)	71.3	76.7	65.8
**Position in hospital hierarchy**			
Junior physicians (n $=$ 88)	66.5	74.8	58.2
Attendings (n $=$ 15)	66.4	72.7	60.0
Senior physicians (n $=$ 45)	67.7	73.0	62.3
Chief physicians (n $=$ 3)	71.3	73.3	69.2
**Practical experience (pe)**			
pe $\leqslant$ 2 years (n $=$ 46)	66.2	76.0	56.5
2 years < pe $\leqslant$ 4 years (n $=$ 37)	66.4	73.8	59.1
4 years < pe $\leqslant$ 12 years (n $=$ 32)	67.9	73.3	62.5
pe > 12 years (n $=$ 42)	67.9	73.0	62.8

### Effectiveness measures

There are three important measurements in medical classification, which are Area Under the Curve (AUC), Sensitivity (SEN), and Specificity (SPE). Mathematically, SEN and SPE can be expressed by true positives (TP), true negatives (TN), false positives (FP) and false negatives (FN) as follows:

5 $$\begin{aligned} sensitivity&= \frac{TP}{TP + FN} \end{aligned}$$

6 $$\begin{aligned} specificity&= \frac{TN}{TN + FP} \end{aligned}$$

In this binary classification, the value returned by the classifier is a real number within [0, 1]. Depending on thresholds, the binary predicted value is either assigned to 0 (negative or nevus) or 1 (positive or melanoma). We can then calculate SEN and SPE as in Formulas (5), (6). The combination of SEN and SPE is measured through a graph with (1 − SPE) on the horizontal axis and SEN on the vertical axis, allowing to draw a corresponding ROC curve as shown in the Figs. 7 and 9. AUC is also represented by the percentage of the area that is under the curve, thus with a value ranging from 0 to 100%.

## Experiment and analysis

In this study, to evaluate the effectiveness of the proposed system, we train the networks with the training dataset and select the final model with minimal loss on the validation dataset. Then we evaluate the performance over the Test-10 dataset and compare it to previous work and to the results of 157 dermatologists on the MClass-D dataset^7^. Our study proposes a new deep architecture with novelty in the introduction of the three following components: custom loss function, custom mini-batch logic, and optimized fully connected layers. The study evaluates and analyzes the results with 3 scenarios as follows: (1) leave both mini-batch logic and loss function unchanged (ORI), (2) customize mini-batch logic as described above and leave the loss function unchanged (BON) and (3) customize both mini-batch logic and loss function (BLF). All three scenarios include a Deep CNN with added fully connected layers as described in Fig. 1. We train the networks with the same number of epochs of 50 and batch size of 32. We choose 50 epochs because during our experiment, we observe that the smaller number of epochs (close to 30) provides maximum SEN and SPE (up to 1.00) for all three scenarios. As we want to keep the weights of the networks stable, we train 20 more epochs and have the number 50. During the training process, we save the model after every epoch with the smallest val_loss, and after completing 50 epochs, we use the model with the smallest val_loss for evaluation. After conducting experiments, we analyze the results with following aspects: (1) the trend of SEN, SPE, and loss during training, (2) the performance over the Test-10 dataset and (3) the performance in comparison to that of the dermatologists on the MClass-D dataset^7^. To allow for the reproducibility of our experiments, the data splits are provided as supplementary material, and the source code of the systems was made publicly available on GitHub at the following address: https://github.com/riseoGit/melanoma-prediction/.

### Training optimizer and learning rate

#### Optimizer

The selection of the optimizer for the network is very important and influences the process of optimizing the weight of the nodes and the performance of the model. In this study, after building the desired network architecture, we experiment with some optimizers such as Adam and SGD^23^ then found that Adam optimizer works better. Thus, we use Adam optimizer for deep experiments. Regarding the initialization of parameters for the optimizer, we use the following settings: lr $=$ 0.0001, beta_1 $=$ 0.9, beta_2 $=$ 0.999, decay $=$ 0.0, epsilon $=$ None and amsgrad $=$ False. The choice of lr will be explained below, while beta_1 and beta_2 are not zero to avoid local minimal.

#### Learning rate

**Figure 3:**
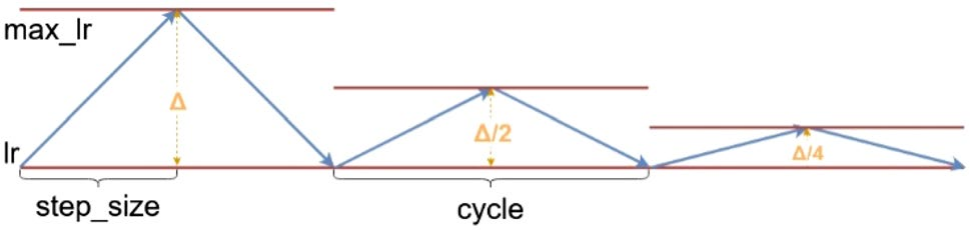
Triangular cycle decreases the cycle amplitude by half after each period, while keeping the base lr constant (this figure was created with Draw.io).

The learning rate (lr) value greatly affects the training of Deep CNN. Usually, when the lr is high in the first epochs, it then decreases in the later epochs, and the model quickly achieves high performance. To do this automatically, we initialize the learning rate for the Adam optimizer to 0.0001, then we use the cyclical learning rate (CLR)^24^ to automatically change the lr in the range from base_lr $=$ 0.0000001 to max_lr $=$ 0.0001 to achieve improved classification performance without the need to tune and to converge in fewer iterations. Besides, we set mode $=$ triangular2 to halve max_lr after each cycle step_size $=$ 4 epochs as depicted in Fig. 3.

### Evaluation of loss, sensitivity and specificity during training and validation

**Figure 4:**
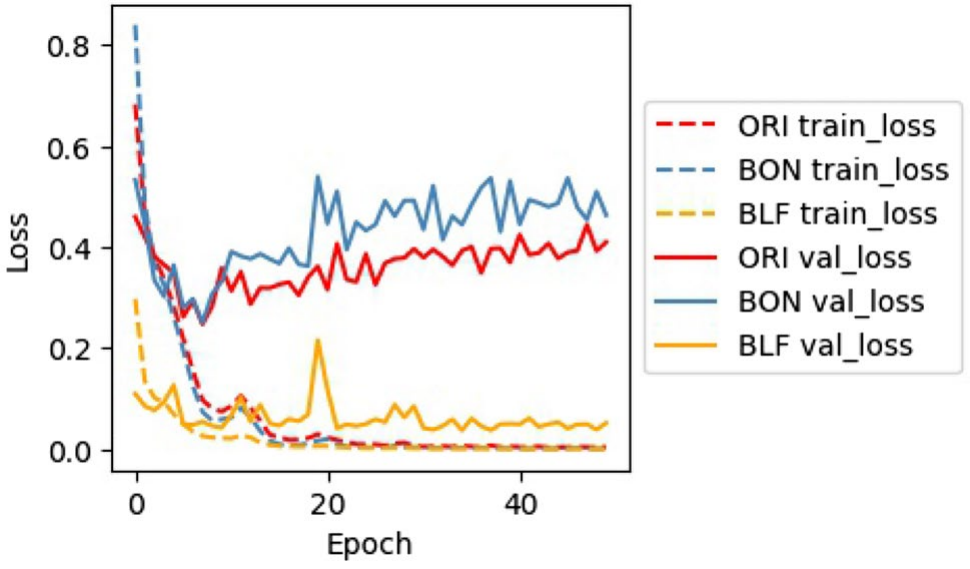
Loss of ORI, BON, and BLF models during training and on the validation dataset.

**Figure 5:**
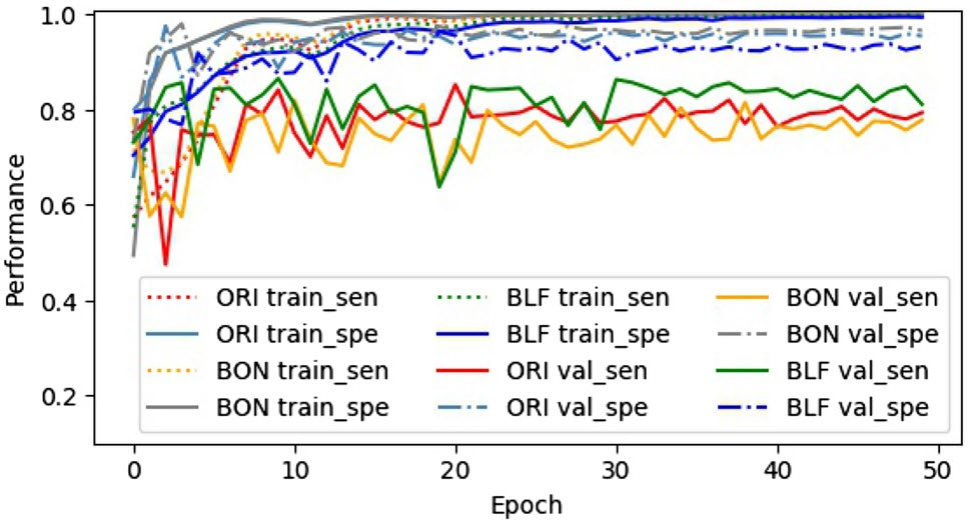
Sensitivity and specificity of ORI, BON, and BLF models during training and on the validation dataset.

In this study, the changes in batch logic, loss function, and fully connected layers aim to optimize the training process. The goal is to increase SEN during training and over the validation dataset after each epoch. Figs. 4 and 5 shows the change of loss, SEN, SPE during training and on the validation dataset.

#### Loss of train and validation

The objective of the training is to establish weights for the nodes of the networks so that the train_loss value is as small as possible. In this study, the loss function of ORI and BON uses the MSE function and has the same unit because they use the same batch size; we therefore can compare both values and trends. In contrast, the loss function of BLF is customized and uses a different unit than MSE, therefore only trends are comparable. As shown in Fig. 4 during training, all three models of ORI, BON, and BLF have substantial different loss with the first 20 epochs. Their stability and convergence reach a minimum value close to 0 from epoch 20. However, only the val_loss of BLF is stable and has a decreasing trend from epoch 20 to 50. On the contrary, BON is unstable from epoch 20 to 50. ORI’s val_loss is always smaller than that of BON, however, the value faces an increasing trend from epoch 20 to 50. This shows that in the process of training, val_loss of BLF is the best, while BON’s val_loss is the worst. This also means that MSE loss function (ORI, BON models) is not adequate, but that our CLF loss function (BLF model) is suitable to train our Deep CNN with imbalanced dataset.

#### Sensitivity and specificity

The ultimate aim of reducing train_loss is also to increase train_sen and train_spe. As the results show in Fig. 5, during training, along with the change of loss from epoch 1 to 30 and 30 to 50, SEN and SPE of ORI, BON, and BLF models have substantial fluctuations when from epoch 1 to 30, then stable and close to 1 from epoch 30 to 50. However, there are differences in val_sen and val_spe. While val_spe of BON is the highest in every epoch, that of BLF is always the smallest. In contrast, val_sen of BLF is always the highest one and that of BON is the lowest in all three models. This suggests that the BLF model has the best balance while the BON model has the poorest one during the training process and on the validation dataset.

By analyzing trends in loss, SEN, and SPE, it can be concluded that the BLF model is the most efficient and most balanced, the ORI model ranks second, and the BON model is the worst. This means that our CLF loss function is suitable for handling the imbalanced class of melanoma when combined with custom mini-batch logic.

### Evaluation performance over the Test-10 dataset

**Figure 6:**
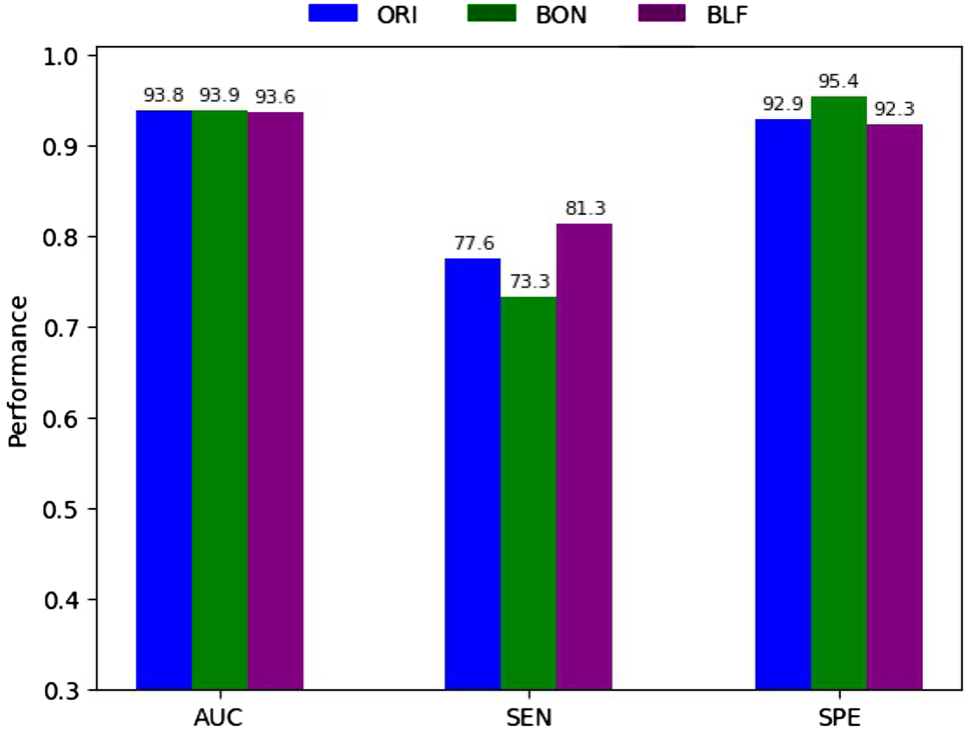
Performance evaluated of three best models of ORI, BON, and BLF over Test-10 dataset using a prediction threshold of 0.5.

**Figure 7:**
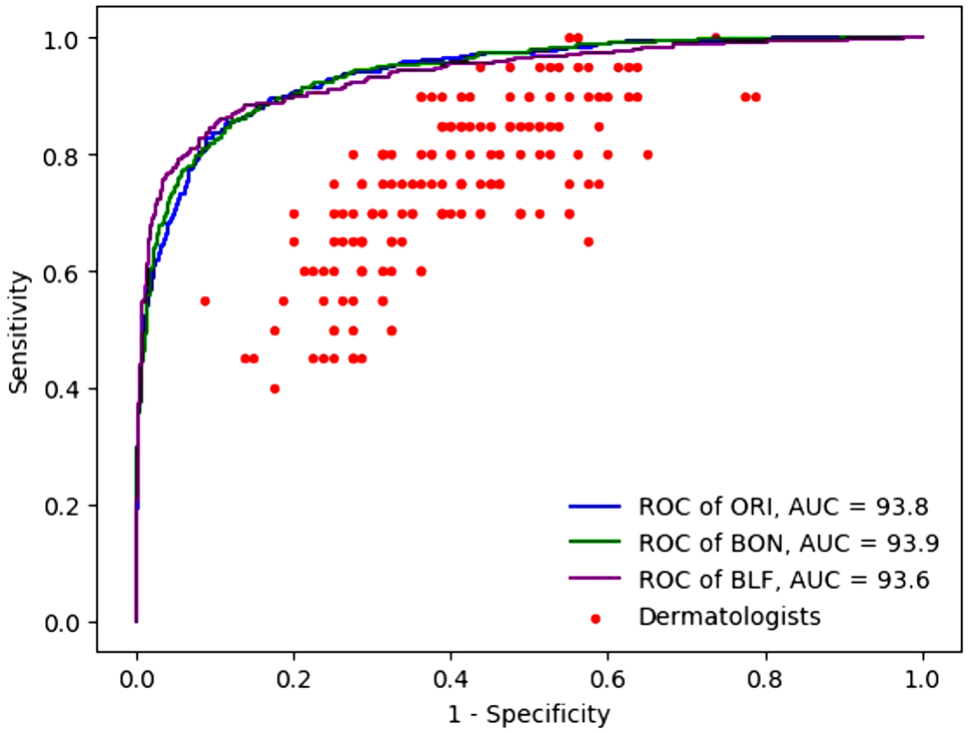
The receiver operating characteristic curves of ORI, BON, and BLF over the Test-10 dataset.

After selecting the best model based on training and validation datasets, we use the Test-10 set (its test data set being composed of 10% of ISIC 2019) to evalutate the model. The evalutation is based on (1) AUC, SEN, and SPE with threshold $=$ 0.5 and (2) the receiver operating characteristic (ROC). Figures 6 and 7 describe these aspects respectively.

#### Performance of the three best models evaluated over Test-10 dataset, using a prediction threshold of 0.5

Figure 6 shows the performance of AUC, SEN, and SPE of the best models of ORI, BON, and BLF. Figure 6 shows that the AUC of all three models is similarly high, the highest being BON with AUC of 93.9 while the lowest is BLF although only 0.3 percentage points lower than BON. However, when considering the balance of SEN and SPE, BLF is the most balanced with respective SEN and SPE scores of 81.3% and 92.3% (difference of 9%), while BON is most unbalanced with 73.6% and 95.4% (difference of 21.8%), and ORI scores 77.6% and 92.9% (difference of 15.3%). BLF’s specificity is only lower than that of ORI by 0.6 percentage points while its SEN is 4.7 percentage points higher. This demonstrates the effectiveness of BLF in maintaining high AUC and SPE while balancing SEN and SPE. The performances of the proposed methods are also much higher than the methods using InceptionV3 whose results are presented in Table 1. In terms of using the same Binary Cross-Entropy loss function, our proposed method using DenseNet169 has an AUC 3.2% higher than InceptionV3’s method when we evaluate the models by the Test-10 dataset. Additionally, our proposed method SEN and SPE performances are higher and more balanced than those of InceptionV3’s method (77.6% and 92.9% vs 61.8% and 96.1%).

Performance evaluation of three best models over the Test-10 dataset using the ROC curve: Fig. 7 illustrates the ROC of the three best models. Three lines in colors blue, green, and purple are respectively representing the ROC curves of ORI, BON, and BLF. In general, the three lines are similar and there are not many differences across the points, reflecting the fact that the differences in AUC are negligible. However, it can be seen that the performance over the Test-10 dataset is higher than the performance of all doctors (with red dots) on the MClass-D dataset^7^ except for two doctors. In addition, BLF shows the exceptional performance when SPE is greater than 80% or SEN is between 70% and 90%. Further detailed analysis of thresholds is provided in Table 5.

**Table 5:** Performances base on Test-10 dataset using different thresholds. In bold, three models achieve high efficiency, especially BLF that reaches SPE near 90% with SEN 85%.

SEN % THRES	SPE %			SPE % THRES	SEN %		
	ORI	BON	BLF		ORI	BON	BLF
100.0	18.9	10.3	2.3	100.0	18.9	20.4	11.8
95.0	68.1	69.3	61.3	95.0	70.0	74.4	77.8
90.0	81.4	80.5	79.9	90.0	83.8	82.4	84.9
**85.0**	**88.6**	**88.8**	**89.9**	**85.0**	**87.3**	**87.3**	**88.4**
80.0	92.0	91.8	93.6	80.0	90.4	90.2	89.8
76.7	93.3	94.1	95.8	69.2	94.2	95.1	93.1
74.1	93.7	95.2	96.7	60.0	96.7	96.0	95.6

Table 5 shows that with Test-10, BLF proves to be the most effective of the three models when SEN is between 74.1% and 90%. All three models have SEN and SPE balanced when SEN is at 85.0% with corresponding SPE of ORI, BON, and BLF respectively at 88.6%, 88.8%, and 89.9%. At this level of SEN, the BLF model remains the best performing model with SPE of 89.9%, 1.3 percentage point better than the worst model of ORI. ORI and BON perform similarly with a difference of only 0.2 percentage points.

### Performance comparison with dermatologists

In this study, to compare the performance of AI models with that of dermatologists, our solutions are evaluated on the MClass-D dataset^7^, the one used to evaluate 157 dermatologists as described in the “Dermatologist performances”. In the present section, we evaluate the work presented through (1) evaluating performance over MClass-D for ORI, BON, and BLF using a prediction threshold of 0.5, and (2) comparing the systems’ performance to that of the dermatologists based on ROC curves.

#### Performance evaluated on the MClass-D data set for the three models using a prediction threshold of 0.5

**Figure 8:**
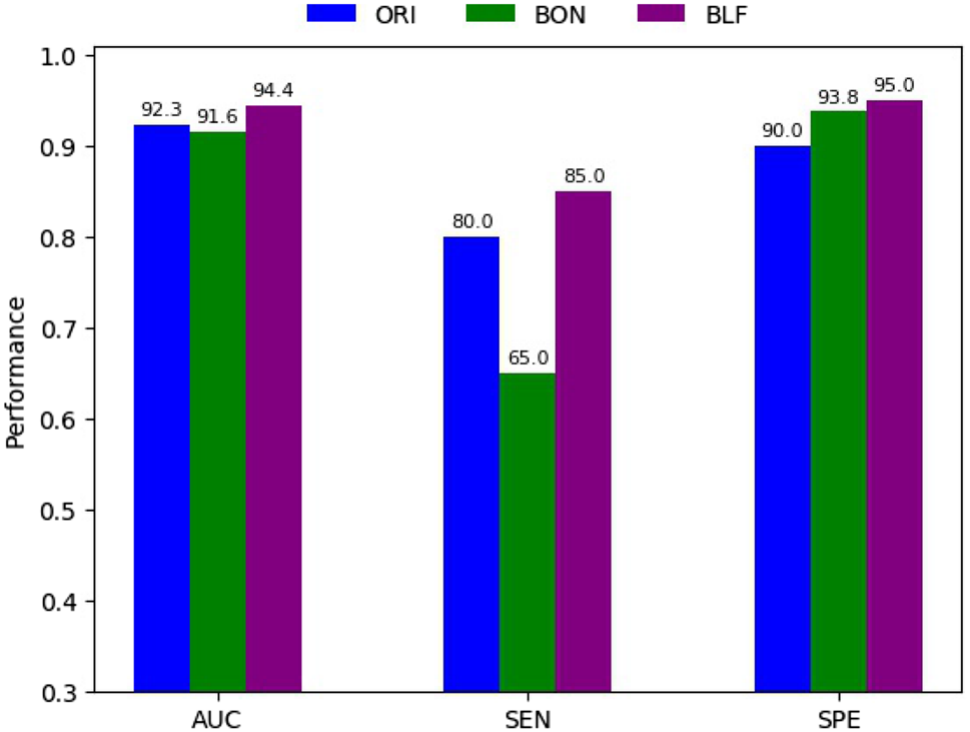
Performance of ORI, BON, and BLF over the MClass-D dataset using a prediction threshold of 0.5.

Figure 8 illustrates the performances of ORI, BON and BLF evaluated on the MClass-D dataset using a prediction threshold of 0.5. In general, BLF gives the best performance in all three measures of AUC, SEN, and SPE with values of 94.4%, 85.0%, and 95.0% respectively, higher than ORI by 2.1, 5.0 and 5.0 percentage points respectively, and even higher than BON (the worst model) by 2.8, 20 and 1.2 percentage points respectively. Considering the SEN and SPE gap, BLF and ORI provide the smallest variation with 10%, while BON produces the worst with 28.8%. However, BLF performs best in terms of SEN (85%) and SPE (95%) 5 percentage points higher than ORI (80% and 90%). These scores provide a new state-of-the-art (SOTA) on the MClass-D dataset. Compared to the study of Young et al.^25^, BLF achieves a 4.0% increase in AUC (94.4% vs 90.4%) on the MClass-D dataset. In terms of the performances over the MClass-D dataset and using the same Binary Cross-Entropy loss function, our proposed methods of DenseNet169 are also much higher than the methods using InceptionV3 whose results are presented in Table 1. The AUC of DenseNet169 model is 8.3% higher than that of InceptionV3. Besides, the SEN and SPE of DenseNet169 are more balanced than those of InceptionV3.

#### Comparison of the systems’ performance to that of dermatologists based on ROC curves

**Figure 9:**
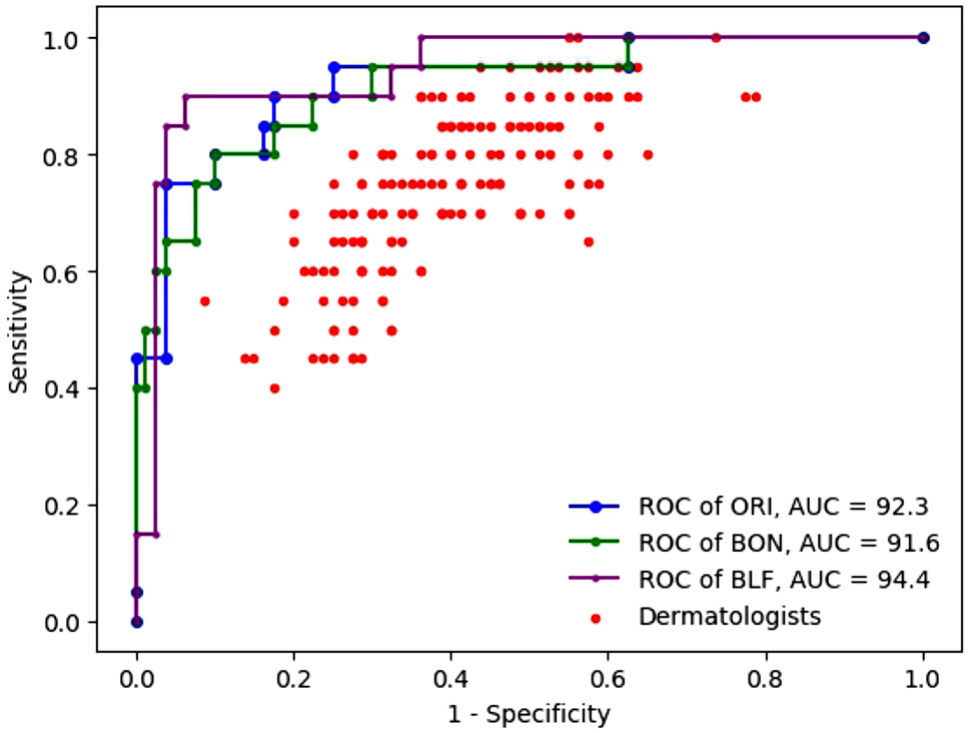
The receiver operating characteristic curves of ORI, BON, and BLF over the MClass-D dataset.

Figure 9 shows the ROC curves of the three best models (in blue, green, and purple, respectively for ORI, BON, and BLF) and the performance of the 157 dermatologists (with red dots). In full details, the AUC of the three models BLF, BON, and ORI respectively stands at 94.4%, 91.6%, and 92.3%, much higher than that of dermatologists (67.1%). There are 154 red points that show the performance (in term of SEN and SPE) of each dermatologist are below the BLF ROC curve, the remaining three points have the SEN of 100% with the highest SPE of 45% on the BLF ROC curve but 18.7% less than that of BLF model (45% vs 63.7%). This means that our BLF model surpasses the performance of every dermatologist. BLF is the best of all three models and has much better performance, being the first system to ever report results better than all of the 157 dermatologists involved in the system, with its AUC of 94.4%. The results of ORI and BON (92.3% and 91.6%) are better than those of 155 of the 157 dermatologists. All three models beat the state of the art, which only outperforms 136 of the dermatologists^26^. A more detailed analysis of the prediction thresholds is provided in Table 6.

In Table 6, SOTA stands the performance of Brinker et al.^26^. As shown in Table 4, the AUC of the dermatologists^7^ and of SOTA^26^ were given for mean SEN and SPE of 74.1% and 60.0%. To ease comparison, Table 6 reminds these scores and provides the corresponding performance of all 3 models. At SEN 74.1%, BLF has a SPE of 97.5%, above the average of dermatologists by 37.3 percentage points, and above SOTA by 11 percentage points. With SPE at 60.0%, the SEN of BLF reaches 100%, while ORI and BON both have SEN of 95%. All three have very high SEN compared to the mean of dermatologists (74.1%) and SOTA (87.5%). Among the group of dermatologists, “chief Physicians” have the highest mean SPE with 69.2% at a mean SEN of 73.3%. With the same SPE of 69.2%, BLF reaches a SEN of 90%, performing 16.7 percentage points higher than this subgroup of the most experienced dermatologists, and 5.5 percentage points higher than SOTA. However, at this level of SPE, ORI and BON actually perform even better in terms of SEN as they reach 95%.

In addition to raising the AUC, the goal of this study is to manage to balance SEN and SPE in order to handle imbalanced dataset. In this respect, BLF proves most effective with SEN and SPE of 90% and 93.8%, surpassing the ORI (85.0% and 83.8%) and BON (85.0% and 82.5%). It can be seen that, at this balance level, BLF is better than ORI and BON. Both the SEN level of 90% and the SPE level of 93.8%, even more so their combination, are very high and applicable in actual practice.

**Table 6:** Performance evaluated over the MClass-D dataset using different thresholds. In bold, three models achieve high efficiency, especially BLF﻿ that reaches SPE 93.8% with SEN 90%.

SEN % THRES	SPE %				SPE % THRES	SEN %			
	ORI	BON	BLF	SOTA		ORI	BON	BLF	SOTA
100.0	37.5	37.5	63.7	–	100.0	45.0	40.0	15.0	–
95.0	75.5	70.0	67.5	–	95.0	75.0	65.0	85.0	–
**90.0**	82.5	77.5	**93.8**	–	90.0	80.0	80.0	90.0	–
**85.0**	**83.8**	**82.5**	**96.2**	**–**	**85.0**	**80.0**	**80.0**	**90.0**	**–**
80.0	90.0	90.0	96.2	–	80.0	90.0	85.0	90.0	–
76.7	90.0	90.0	96.2	–	69.2	95.0	95.0	90.0	84.5
74.1	96.2	92.5	97.5	86.5	60.0	95.0	95.0	100.0	87.5

## Discussion

Our proposed binary melanoma classification system, which is based on deep CNN with optimization in batch logic, loss function, and fully connected layers, is trained with the latest public datasets. It is the first to outperform all 157 dermatologists with qualifications from different hospitals, while the latest best-performing publication with the same dataset only outperforms 136 dermatologists^26^. Besides this head-to-head comparison, our solution outperforms both state-of-the-art and dermatologists on the general. First of all, the AUC of our proposed solution reached 94.4%, 27.3 percentage points better than the average of all the dermatologists, and 23.3 percentage points better than that of private practice and chief physicians. This study, therefore, provides new state-of-the-art AUC for binary melanoma classification on the MClass-D dataset. Secondly, with a prediction threshold of 0.5, our AI model achieves relatively balanced SEN and SPE at 85.0% and 95.0% respectively, much higher than the average of the physicians, which lies at 74.1% and 60.0% respectively. Lastly, since the output is a real number in the range of [0, 1], adjusting the threshold provides a simple and efficient way to put a stronger emphasis on either SEN or SPE. This is done as a post-processing step, requiring milliseconds of processing time on a simple device. BLF remains the best model with a threshold of 0.40858, with respective SEN and SPE of 90.0% and 93.8%. This is the best and most balanced indicator.

BLF is the best-performing model that is built with our optimized Deep CNN architecture and both of our proposed custom mini-batch logic and custom loss function. Two other models are also introduced, allowing us to measure the impact of our three additions to the state-of-the-art. ORI is a model built with the optimized Deep CNN architecture but neither with the loss function nor the batch logic. BON is with the Deep CNN architecture and the batch logic, but without the proposed loss function. Both of these models are also very effective, showing the key role of our optimized architecture of Deep CNN. They outperformed 155 dermatologists and respectively achieved AUC 2.1 and 2.8 percentage points lower than that of BLF. In the evaluation on the Test-10 dataset, the AUC of ORI even exceeds that of BLF. This means that the proposed system architecture of using DenseNet169 with optimized fully connected layers is effective for melanoma prediction.

The best-performance of BLF shows that an effective method to balance the two indicators of SEN and SPE in binary melanoma classification can be implemented by changing the loss function as detailed in Formula (4), fine-turning the parameters *a* and *b* so as to fit the datasets relevant to the adequate medical image classification tasks. We indeed believe that this result can be generalized to other applications. In the case of binary melanoma classification, the parameter values *a* $=$ 0.5 and *b* $=$ 1.0 produce the best results. In addition, the custom mini-batch logic also affects the training process as it both helps to balance the indicators of SEN and SPE and to provide fast convergence.

Binary melanoma classification requires the reforming fully connected layers deep enough to have the ability to learn on the large dataset. The combination of many fully connected hidden layers with custom mini-batch logic and custom loss function gives excellent results and exceeds the humans’ accuracy and the previous state-of-the-art for this.

## Conclusions

In this study, we propose a new approach to binary melanoma classification by reforming fully connected layers and customizing mini-batch logic and loss function. The major results of this research are listed below:

1. Our proposed system achieves state-of-the-art AUC of 94.4% at higher SEN of 85.0% and specificity of 95.0% and outperforms all of the 157 dermatologists on the MClass-D dataset^7^. The balanced SEN and SPE is 90.0% and 93.8% when using a prediction threshold of 0.40858 is the state-of-the-art performance on the MClass-D dataset.
2. At SEN 74.1%, the proposed method has a specificity of 97.5%, exceeding the average of dermatologists by 37.3% and current SOTA by 11%^11^. Moreover, with SPE 60.0%, the proposed method reached 100% SEN, 25.9% higher than the mean of dermatologists, and 12.5% the current SOTA.
3. Proposed custom loss function combined with custom mini-batch logic effectively handles the imbalance class, improves the balance of SEN and SPE, and obtains the best performances in terms of AUC, SEN, and SPE.

The study is not only significant in terms of AUC, SEN, and SPE for binary melanoma classification. The research results also show that customizing batch logic, loss function, and reforming fully connected layers are very important features for a high-performing model to classify medical images. We intend to develop more intensive studies on custom loss function and custom fully connected layers, apply not only to melanoma classification but also to other medical image classification tasks.

## Supplementary Information

Supplementary Information 1.

Supplementary Information 2.

Supplementary Information 3.

Supplementary Information 4.
